# Supplementary material for: Ligand‐induced ubiquitination regulates endocytosis and homeostasis of the ERECTA receptor kinase for stomatal development
Source: New Phytol. 2026 Apr 3;250(5):3167–81. doi: 10.1111/nph.71151 (PMC13150308; doi:10.1111/nph.71151)
Supplement: Supplementary file 2 — Fig. S1 Ubiquitination‐deficient ERECTA overly promotes pedicel growth, related to Fig. 2. Fig. S2 Expression level of ERECTA in ERECTA‐FLAG er, ERECTA 3KR ‐FLAG er, ERECTA‐YFP er, ERECTA 3KR ‐YFP er, related to Fig. 2. Fig. S3 The predicted ubiquitination sites are required for polyubiquitination of ERECTA by PUB31, related to Fig. 3. Fig. S4 K63‐linked ubiquitination is required for eventual vacuolar degradation of ERECTA, related to Fig. 5. Fig. S5 The ubc35 ubc36 double mutant exhibits normal stomatal development and pedicel elongation. Table S1 List of plasmids used in this study. Table S2 List of primers used in this study. Please note: Wiley is not responsible for the content or functionality of any Supporting Information supplied by the authors. Any queries (other than missing material) should be directed to the New Phytologist Central Office. [file NPH-250-3167-s002.pdf]

New Phytologist

***New Phytologist* Supporting Information**

Article title: Ligand-induced ubiquitination regulates endocytosis and homeostasis of the ERECTA receptor kinase for stomatal development

Authors: Liangliang Chen1,2*, Minh Huy Vu1,2*, Pengfei Bai1,2 Alicia M. Cochran1,2, Crystal F. Ying2, and Keiko U. Torii1,2

Article acceptance date: 09 March 2026

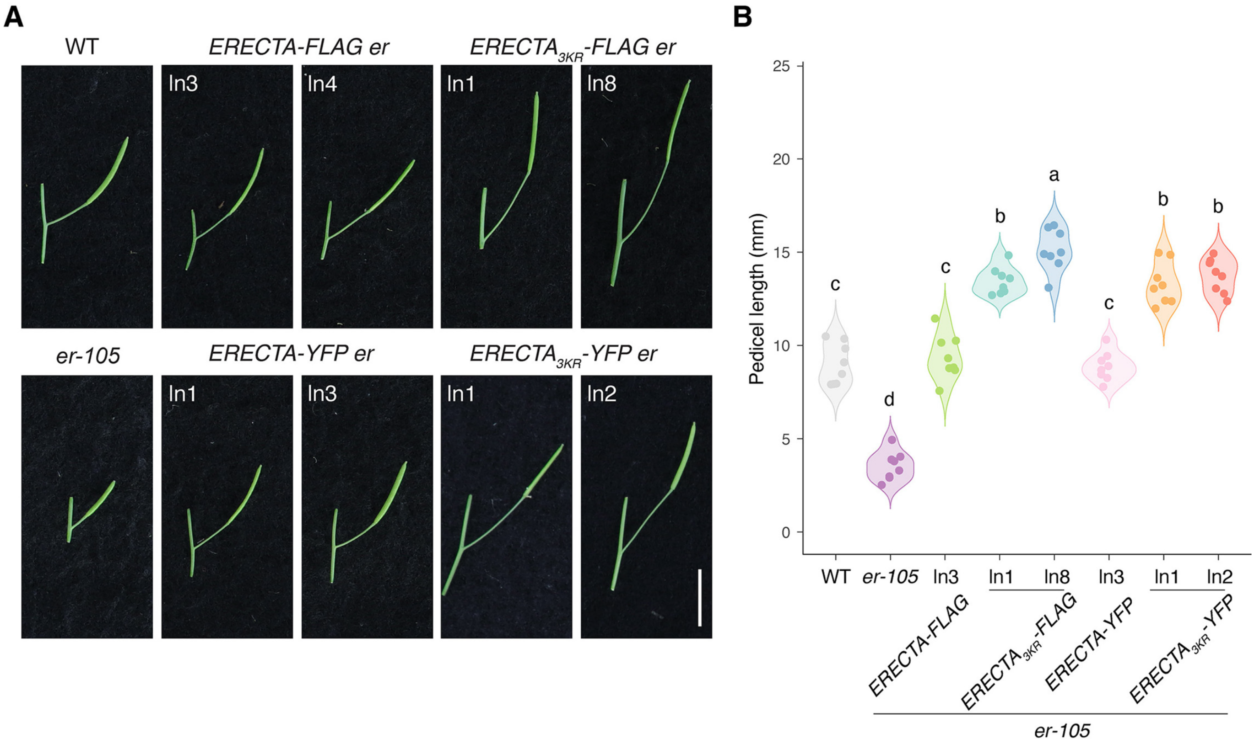

Fig. S1 Ubiquitination-deficient ERECTA overly promotes pedicel growth, related to Fig. 2 **(A)** Representative pedicels and mature siliques of WT, *er-105* (*er*), *ERECTA-FLAG er*, *ERECTA*$_{3KR}$*-FLAG er*, *ERECTA-YFP er*, *ERECTA*$_{3KR}$*-YFP er* plants. For each transgenic construct, two representative lines were subjected to analysis. Images were taken under the same magnification. (Scale bar, 10 mm.) **(B)** Morphometric analysis of pedicel length from each genotype. 6-wk-old mature pedicels (n = 8 plants) were measured. Data are presented as violin plots where the width represents data density and all individual data points are displayed to show the full distribution. One-way ANOVA followed by Tukey's HSD test was performed, and statistically different groups are labeled with distinct letters (e.g., a, b, c, d), while groups labeled with the same letter are not statistically different from each other ($P < 0.05$).

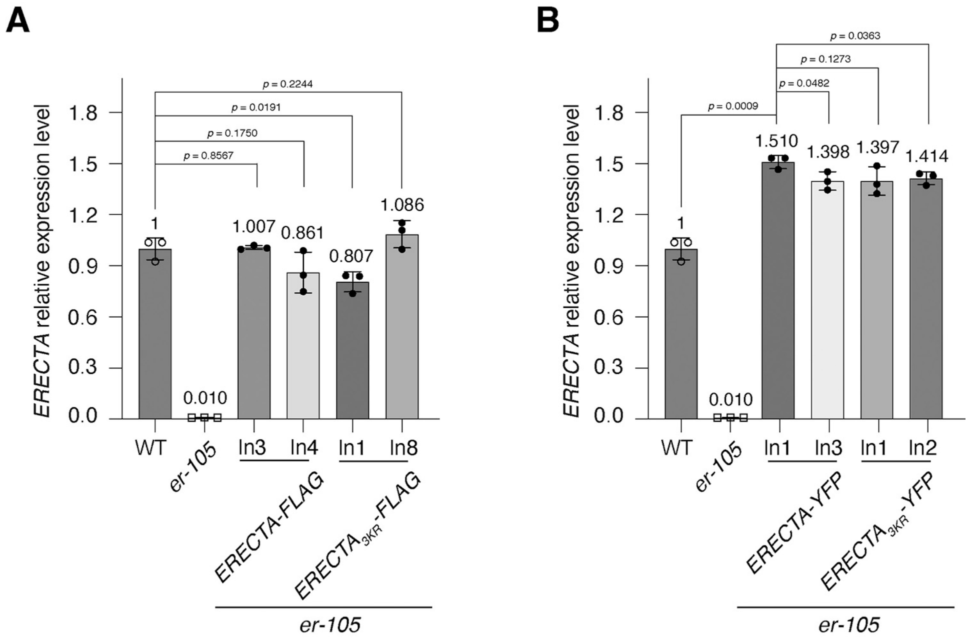

Fig. S2 Expression level of *ERECTA* in *ERECTA-FLAG er*, *ERECTA$_{3KR}$-FLAG er*, *ERECTA-YFP er*, *ERECTA$_{3KR}$-YFP er*, related to Fig. 2

(A) RT-qPCR analysis of *ERECTA* in WT, *er-105* (*er*), *ERECTA-FLAG er*, *ERECTA$_{3KR}$-FLAG er.* Transcript levels were normalized against *ACTIN* (*ACT2*) and adjusted to 1 for wild type. Bars, mean values of three technical replicates. Welch's unpaired t-tests were performed, and the corresponding p-values were labeled on the plots. Bars represent mean ± SD.

(B) RT-qPCR analysis of *ERECTA* in WT, *er-105* (*er*), *ERECTA-YFP er*, *ERECTA$_{3KR}$-YFP er.* Transcript levels were normalized against *ACT2* and adjusted to 1 for wild type. Bars, mean values of three technical replicates. Welch's unpaired t-tests were performed, and the corresponding p-values were labeled on the plots. Bars represent mean ± SD.

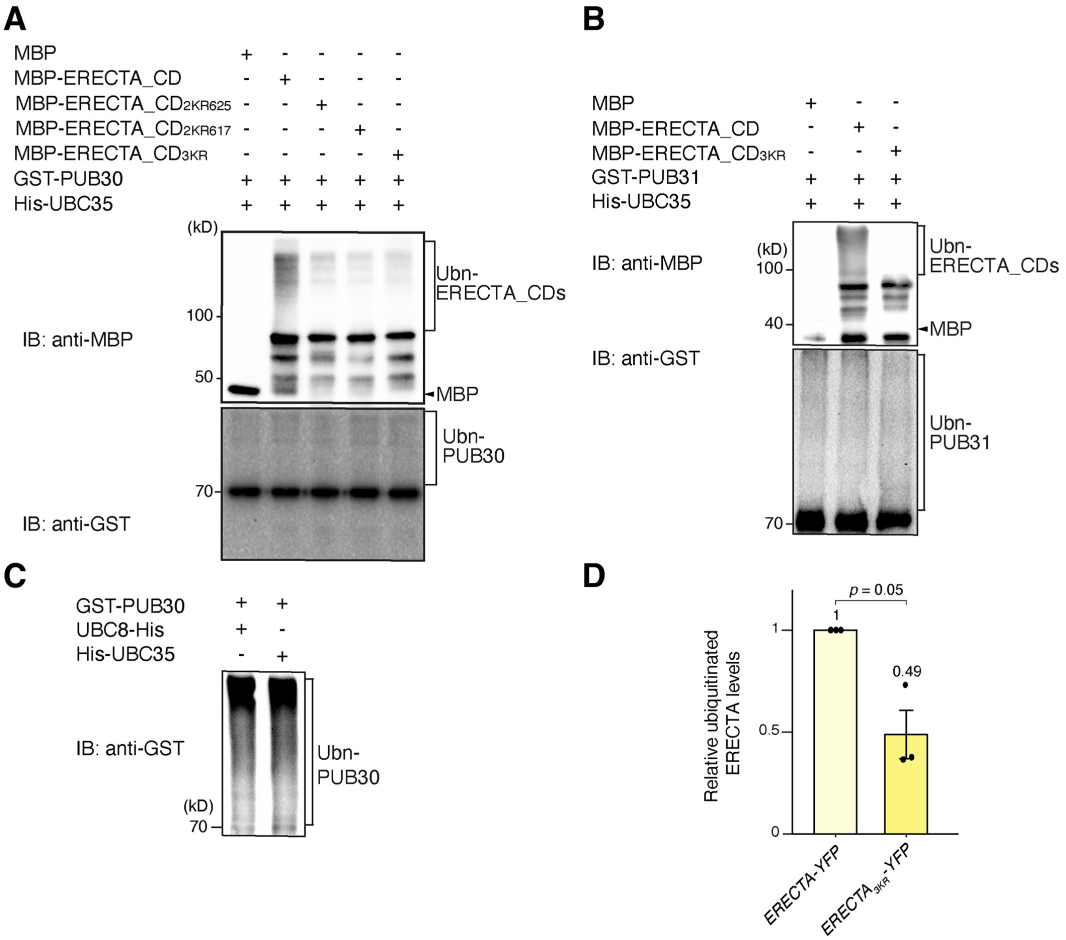

Fig. S3 The predicted ubiquitination sites are required for polyubiquitination of ERECTA by PUB31, related to Fig. 3 **(A)** Lysines K617, K625, and K668 are required for the ubiquitination of ERECTA by PUB30 in vitro. The ubiquitination of MBP-ERECTA_CD, MBP-ERECTA_CD$_{2KR617}$, MBP-ERECTA_CD$_{2KR625}$ or MBP-ERECTA_CD$_{3KR}$ was carried out by using GST-fused PUB30 as the E3 ligase, His-fused AtUBA1 as E1 activating enzyme, and His-fused UBC35 as E2 conjugating enzyme. **(B)** Lysines K617, K625, and K668 are required for the ubiquitination of ERECTA by PUB31 *in vitro*. The ubiquitination of MBP-ERECTA_CD or MBP-ERECTA_CD$_{3KR}$ was carried out by using GST-fused PUB31 as the E3 ligase, His-fused AtUBA1 as E1 activating enzyme, and His-fused UBC35 as E2 conjugating enzyme. **(C)** UBC8 and UBC35 exhibit comparable activity for autophosphorylation of PUB30. The auto-ubiquitination of GST-PUB30 was performed using His-fused AtUBA1 as E1 activating enzyme, and His-fused UBC8 or UBC35 as E2 conjugating enzyme. **(D)** Quantification of K63 linked-polyubiquitination level of ERECTA-YFP and ERECTA$_{3KR}$-YFP immunoprecipates in Fig. 3B (n = 3 biological replicates). Welch's unpaired t-tests were performed, and the corresponding p-values were labeled on the plots. Bars represent mean ± SD.

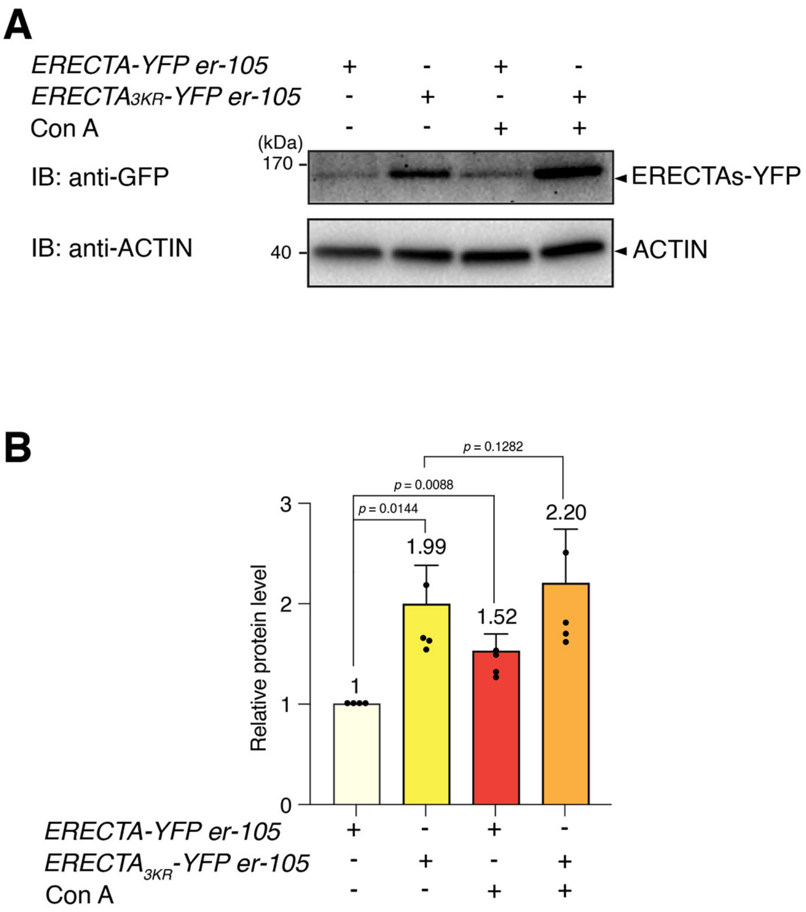

Fig. S4 K63-linked ubiquitination is required for eventual vacuolar degradation of ERECTA, related to Fig. 5

(A) Protein accumulation in *ERECTA-YFP er* and *ERECTA$_{3KR}$-YFP er*, in the absence and presence of the vacuolar ATPase inhibitor Concanamycin A (Con A). Total proteins were isolated from 5-day-old seedlings and probed by an α-GFP antibody. The protein inputs were equilibrated using α-Actin antibodies.

(B) Quantification of protein abundance (ERECTA/Actin and *ERECTA$_{3KR}$*/Actin) (n = 4 biological replicates) in the absence and presence of Con A. Welch's unpaired t-tests were performed, and the corresponding p-values were labeled on the plots. Bars represent mean ± SD.

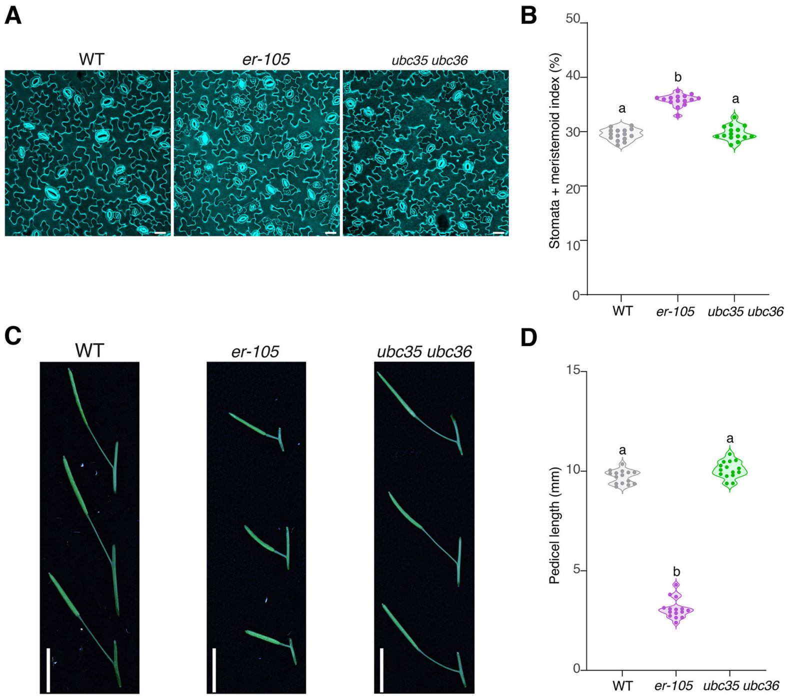

Fig. S5 The *ubc35 ubc36* double mutant exhibits normal stomatal development and pedicel elongation.

(A) Representative confocal microscopy of cotyledon abaxial epidermal from 7-day-old wild type (WT), *er-105* and *ubc35 ubc36* plants. The same representative transgenic lines were observed. Images were taken under the same magnification. Scale bar: 25 μm.

(B) Quantitative analysis. Stomata + meristemoid index (number of stomata and meristemoid per 100 epidermal cells) of the cotyledon abaxial epidermis from 7-day-old seedlings of respective genotypes (n = 15 seedlings). Data are presented as violin plots where the width represents data density and all individual data points are displayed to show the full distribution. One-way ANOVA followed by Tukey's HSD test was performed, and statistically different groups are labeled with distinct letters (e.g., a, b, c, d), while groups labeled with the same letter are not statistically different from each other ($P < 0.05$).

(C) Representative pedicels and mature siliques of WT, *er-105* and *ubc35 ubc36* plants. Images were taken under the same magnification. (Scale bar, 10 mm.)

(D) Morphometric analysis of pedicel length from each genotype. 6-wk-old mature pedicels (n = 15 plants) were measured. Data are presented as violin plots where the width represents data density and all individual data points are displayed to show the full distribution. One-way ANOVA followed by Tukey's HSD test was performed, and statistically different groups are labeled with distinct letters (e.g., a, b, c, d), while groups labeled with the same letter are not statistically different from each other ($P < 0.05$).

New Phytologist

Table S1 List of plasmids used in this study

Plasmid	Insert	Backbone vector	Antibiotics	Purpose	Notes
pCLL202	ERECTApro::gERECTA	R4pGWB410	Spec	For plant transformation	Chen et al., (2025)
pCLL201	ERECTApro::gERECTA$_{K617RK668R}$	R4pGWB410	Spec	For plant transformation	This study
pCLL200	ERECTApro::gERECTA$_{K625RK668R}$	R4pGWB410	Spec	For plant transformation	This study
pCLL284	ERECTApro::gERECTA$_{K668R}$	R4pGWB410	Spec	For plant transformation	This study
pCLL287	ERECTApro::gERECTA$_{K617RK625RK668R}$	R4pGWB410	Spec	For plant transformation	This study
pCLL283	ERECTApro::gERECTA	R4pGWB540	Spec	For plant transformation	This study
pCLL288	ERECTApro::gERECTA$_{K617RK625RK668R}$	R4pGWB540	Spec	For plant transformation	This study
pJSL104	gERECTA	pENTR/D-topo	Kan	As entry clone	Chen et al., (2025)
pLGR1	ERECTA promoter	pENTR/5'-topo	Kan	As entry clone	Lee et al. (2012)
pCLL199	ERECTApro::gERECTA$_{K617RK668R}$	pENTR/D-topo	Kan	As entry clone	This study
pCLL198	ERECTApro::gERECTA$_{K625RK668R}$	pENTR/D-topo	Kan	As entry clone	This study
pCLL286	ERECTApro::gERECTA$_{K617RK625RK668R}$	pENTR/D-topo	Kan	As entry clone	This study
pJA51	ERECTA_CD	pMAL-c2	Amp	For recombination expression	Chen et al., (2023)
pCLL193	ERECTA_CD$_{K625RK668R}$	pMAL-c2	Amp	For recombination expression	This study
pCLL194	ERECTA_CD$_{K617RK668R}$	pMAL-c2	Amp	For recombination expression	This study
pCLL285	ERECTA_CD$_{K617RK625RK668R}$	pMAL-c2	Amp	For recombination expression	This study
pCLL107	PUB30-CDS	pGEX4T-1	Amp	For recombination expression	Chen et al., (2023)
pCLL109	PUB31-CDS	pGEX4T-1	Amp	For recombination expression	Chen et al., (2023)
pJSL68	mature EPF2	pBADgIII	Amp	For recombination expression	Lee et al. (2012)
pJSL79	mature EPFL6	pBADgIII	Amp	For recombination expression	Uchida et al. (2012)
pCLL148	PUB30 without stop codon (1-1344bp)-1xMYC	pHBT	Amp	For protoplast transfection	Chen et al., (2023)
pJSL84	ERECTA without stop codon (1-5526bp)-2xHA	pHBT	Amp	For protoplast transfection	Lee et al. (2012)
pCLL290	gERECTA$_{K617RK625RK668R}$ without stop codon (1-5526bp)-2xHA	pHBT	Amp	For protoplast transfection	This study
pJSL85	ERECTA without stop codon (1-5526bp)-2xFLAG	pHBT	Amp	For protoplast transfection	Chen et al., (2025)

Table S1 List of plasmids used in this study (continued)

Plasmid	Insert	Backbone vector	Antibiotics	Purpose	Notes
pCLL289	gERECTAK617RK625RK668R without stop codon (1-5526bp)-2xFLAG	pHBT	Amp	For protoplast transfection	This study
NA	UBA1	pET22b	Amp	For recombination expression	Zhou et al. (2018)
NA	UBC8	pET22b	Amp	For recombination expression	Zhou et al. (2018)
NA	UBC35	pDEST17	Amp	For recombination expression	Turek et al., 2018
NA	FLAG-UBQ	pHBT	Amp	For protoplast transfection	Zhou et al. (2018)

New Phytologist

Table S2 List of primers used in this study

Gene names	Primer names	Sequences (5' to 3')	Note
**For Constructs**			
ERECTA	gERECTA-Dtopo-LP	caccATGGCTCTGTTTAGAGATATTGT	for gERECTAs-D-TOPO
	gERECTA-Dtopo-RP	CTCACTGTTCTGAGAAATAACTTG	for gERECTAs-D-TOPO
	ERECTA-K668R-LP2	ACAAATGTGTTTTGAgGAATTGTAAACCG	for introducing K668R to gERECTA & ERECTA_CD
	ERECTA-K668R-RP1	CGGTTTACAATTCcTCAAAACACATTTGT	for introducing K668R to gERECTA & ERECTA_CD
	ERECTA-K617R-LP2	GATGGATCACTTGACAgACCAGgtctactc	for introducing K617R to gERECTA
	ERECTA-K617R-RP1	gagtagacCTGGTcTGTCAAGTGATCCATC	for introducing K617R to gERECTA
	ERECTA-K625R-LP2	AACTTATTCGACACCGcgGCTCGTCATCCT TCATAT	for introducing K625R to gERECTA
	ERECTA-K625R-RP1	ATATGAAGGATGACGAGCcgCGGTGTCGAA TAAGTT	for introducing K625R to gERECTA
	MBP-ERECTA_CD-BamH1-LP	GAAGGATTTCAGAATTCGGATCCGCTTGC CGACCGCATAATCCTC	for MBP-ERECTA_CDs
	MBP-ERECTA_CD-Sal1-RP	GCCAAGCTTGCCTGCAGGTCGACCTACTC ACTGTTCTGAGAAAT	for MBP-ER_CDs
	ERECTA_CD-K617R-LP2	ATCACTTGACAgACCAGTAACTTATTCG	for introducing K617R to ERECTA_CD
	ERECTA_CD-K617R-RP1	CGAATAAGTTACTGGTcTGTCAAGTGAT	for introducing K617R to ERECTA_CD
	ERECTA_CD-K625R-LP2	ATTCGACACCGcgGCTCGTC	for introducing K625R to ERECTA_CD
	ERECTA_CD-K625R-RP1	GACGAGCcgCGGTGTCGAAT	for introducing K625R to ERECTA_CD
	pHBT-ERECTA-BamH1-F	GCTCTCGGCTCCCTCTCCCCTTGCTCCGT GGATCCATGGCTCTGTTTAGAGATATTGT	for pHBT-ERECTAs-HA/FLAG
	pHBT-FLAG-ERECTA-Stu1-R	GTCACTTGTCATCGTCGTCCTTGTAGTCAG AAGGCCTCTCACTGTTCTGAGAAATAACTT	for pHBT-ERECTAs-FLAG
**For genotyping**			
ERECTA	ERg2248	AAGAAGTCATTCAAAGATGTGA	ERg2248+ERg3016rc for *ERECTA* WT band
	ERg3016rc	AGAATTTCCAGGTTTGGAATCTGT	
	er-105rc	AGCTGACTATACCCGATACTGA	ERg2248+er-105rc for *ERECTA* insert band
**For q-RT PCR**			
	ACT2-F	TCATCTTCTTCCGCTCTTTCTT	
	ACT2-R	AATCCAGCCTTCACCATACC	
	qERECTA-F	ACAGCTTAAATGTTCATGG	
	qERECTA-R	GGACCTTTGATATTGTTGC	
